# Vegetarian Dietary Patterns and Metabolic Health: Insights from Dietary Quality, Cardiometabolic Outcomes, and Food Environments

**DOI:** 10.3390/nu18213661

**Published:** 2026-11-04

**Authors:** Evelien Mertens, Peter Deriemaeker

**Affiliations:** 1 Research Centre for Preventive Health Innovation, Erasmushogeschool Brussel, 1090 Brussels, Belgium; 2 Nutrition and Dietetics Program, EhB School of Health & Environment, Erasmushogeschool Brussel, 1090 Brussels, Belgium; peter.deriemaeker@ehb.be; 3 Department of Movement and Sport Sciences, Faculty of Physical Education and Physiotherapy, Vrije Universiteit Brussel, 1050 Brussels, Belgium

## 1. Introduction

Metabolic syndrome (MetS), characterized by a combination of abdominal obesity, impaired glucose metabolism, dyslipidemia, and elevated blood pressure, represents a major public health challenge due to its association with cardiovascular disease and type 2 diabetes. Dietary modification remains a cornerstone of prevention and management, with growing interest in vegetarian and plant-based dietary patterns as potentially beneficial approaches. However, the evidence assembled in this Special Issue primarily concerns cardiometabolic risk factors, cardiovascular outcomes, and related metabolic conditions rather than directly assessing the incidence or prevention of MetS as a defined clinical syndrome. Evidence syntheses specifically addressing vegetarian diets and plant-based diet indices have reported heterogeneous associations with MetS, underscoring the need to distinguish dietary definitions and study designs [1,2].

Terminology is important in this context. Vegetarian dietary patterns exclude meat and fish but may include eggs and dairy products; vegan dietary patterns exclude all animal-derived foods. The broader term plant-based encompasses dietary patterns emphasizing plant foods to varying degrees and does not necessarily imply complete exclusion of animal-derived foods. Moreover, these patterns are heterogeneous, ranging from diets rich in vegetables, fruits, whole grains, legumes, and nuts to those containing substantial amounts of refined carbohydrates and other less nutritious plant-derived foods. Their health effects therefore cannot be inferred from the label alone. Prospective cohort findings also suggest that the healthfulness of plant foods, rather than plant-food predominance alone, is important for coronary outcomes [3,4].

The Special Issue “Vegetarian Dietary Patterns in the Prevention of Metabolic Syndrome” brings together six contributions addressing different dimensions of plant-based nutrition and metabolic health. These include prospective epidemiological studies, exploratory dietary intervention analyses, a narrative review of biological mechanisms, and an investigation of food provision in healthcare settings. Taken together, they inform the wider context of MetS prevention without collectively establishing direct effects on MetS incidence.

## 2. Key Contributions of the Special Issue

### 2.1. Plant-Based Dietary Quality and Cardiovascular Health

Two studies in this Special Issue highlight the importance of considering food quality and preparation methods when investigating associations between plant-derived foods and cardiovascular outcomes.

Park et al. (Contribution 1) examined the associations between overall, healthful, and unhealthful plant-based diet indices and cardiovascular disease incidence in a prospective cohort of 10,030 middle-aged Korean adults. These indices quantify relative consumption of plant and animal food groups and do not classify participants as necessarily vegetarian or vegan. None of the indices was significantly associated with overall cardiovascular disease risk. However, higher adherence to an unhealthful plant-based diet was associated with an increased risk of coronary heart disease (hazard ratio: 1.62; 95% confidence interval: 1.12–2.33), whereas no significant associations were observed for the overall or healthful plant-based diet indices. These findings emphasize that greater consumption of plant-derived foods does not necessarily translate into improved cardiovascular outcomes.

Similarly, Djousse et al. (Contribution 2) investigated potato consumption and the incidence of cardiovascular disease and hypertension using harmonized data from prospective cohorts. Total potato consumption was not significantly associated with either outcome. However, fried potato consumption was associated with a modestly higher risk of hypertension, while no corresponding association was observed for cardiovascular disease. This food-specific analysis does not assess adherence to vegetarian diets, but illustrates why preparation methods matter when characterizing plant-derived foods.

Together, these studies reinforce the need to move beyond a simple plant-versus-animal distinction and consider the nutritional composition, processing, and preparation of foods when assessing the relationship between plant-based diets and cardiometabolic health. This interpretation is consistent with research distinguishing healthful from unhealthful plant-based diet indices [3].

### 2.2. Inflammation, Thrombosis, and Metabolic Dysfunction

Beyond conventional cardiometabolic risk factors, the potential effects of plant-based dietary patterns on inflammatory and thrombotic pathways warrant further investigation.

In their narrative review, Jafarnezhad et al. (Contribution 3) explored the complex relationships between metabolic syndrome, chronic low-grade inflammation, coagulation abnormalities, and vascular health. The authors considered vegan and other plant-based dietary patterns, which should not be treated as interchangeable exposures, and discussed potential influences of fiber, fatty acid composition, and bioactive compounds. Although the literature suggests potentially beneficial effects, mechanistic hypotheses require further clinical confirmation.

Cerabino et al. (Contribution 4) investigated the relationship between follistatin and plasminogen activator inhibitor-1 (PAI-1) in 44 individuals with obesity and metabolic dysfunction-associated steatotic liver disease (MASLD). After a two-month intervention involving increased vegetable consumption and reduced carbohydrate portions, PAI-1 concentrations decreased significantly, whereas follistatin remained relatively stable. Higher follistatin concentrations were independently associated with lower PAI-1 levels after the intervention. The exploratory results do not establish a causal regulatory role for follistatin. Importantly, the participants and dietary intervention appear to be the same as those reported in the subsequent analysis by Cerabino et al. (Contribution 5); these publications should therefore be understood as complementary analyses of one pilot intervention, not independent replications.

### 2.3. Practical Dietary Strategies for Metabolic Health

In a complementary report on the same apparent cohort of 44 adults with obesity and MASLD, Cerabino et al. (Contribution 5) evaluated a single-food substitution strategy: replacing one daily serving of carbohydrate-rich foods with 200 g of cruciferous vegetables over two months, without prescribed caloric restriction. This intervention increased the vegetable component of the diet but did not constitute a prescribed vegetarian or vegan dietary pattern.

The intervention was associated with reductions in fat mass and improvements in several metabolic and hepatic parameters. These findings suggest that targeted food substitutions may represent a practical approach to improving dietary composition in individuals with metabolic dysfunction.

However, the absence of a control group, the relatively small sample size, and the short intervention period limit causal interpretation and generalizability. Furthermore, the observed improvements cannot be attributed exclusively to specific properties of cruciferous vegetables, as changes in overall energy intake and dietary composition may also have contributed.

Nevertheless, this study highlights the relevance of investigating feasible dietary modifications that may be easier to implement than more complex dietary interventions.

### 2.4. Translating Evidence into Supportive Food Environments

While understanding the physiological effects of plant-based dietary patterns is essential, their successful implementation also depends on the availability and accessibility of nutritionally adequate food choices.

Mertens et al. (Contribution 6) investigated the availability of vegetarian and fully plant-based (vegan) meal options in Dutch-speaking Belgian hospitals. Their cross-sectional study identified substantial gaps in plant-based meal provision, particularly regarding protein-rich foods such as legumes and minimally processed meat alternatives. Knowledge gaps among food service staff and organizational barriers were also reported. Here, the distinction between vegetarian meals, which may contain eggs or dairy, and fully plant-based meals is particularly relevant to nutritional planning.

These findings underline the importance of addressing institutional food environments alongside individual dietary behavior. Healthcare institutions may provide opportunities to support healthier and more sustainable dietary patterns, but the provision of plant-based meals requires attention to nutritional adequacy, practical feasibility, and staff expertise.

Although this study did not measure MetS incidence or cardiometabolic outcomes, it contributes an implementation perspective to the broader discussion of dietary quality and disease prevention.

## 3. Knowledge Gaps and Future Research Directions

The contributions to this Special Issue highlight several priorities for future research.

First, greater consistency in defining and measuring dietary exposures is needed. Vegetarian and vegan dietary patterns should be distinguished from broader plant-based indices, which can also describe omnivorous diets with a high proportion of plant foods. Food-group quality, processing, and preparation should be assessed separately where possible; processing categories alone do not capture nutritional quality.

Second, more high-quality intervention studies are required to establish causal relationships between dietary modifications and metabolic outcomes, including directly measured MetS incidence or changes in its diagnostic components. The two reports by Cerabino et al. (Contributions 4 and 5) provide complementary outcomes from what appears to be a single, small, uncontrolled pilot intervention, rather than two independent demonstrations of efficacy. Larger randomized controlled trials with longer follow-up could clarify the effectiveness and sustainability of such substitutions.

Third, further investigation of inflammatory, metabolic, and thrombotic biomarkers may improve our understanding of the biological mechanisms underlying the associations between plant-based nutrition and cardiometabolic health. Particular attention should be given to distinguishing mechanistic hypotheses from clinically demonstrated effects.

Finally, research should increasingly address the translation of nutritional evidence into everyday practice. Institutional settings, including hospitals, offer opportunities to facilitate healthier dietary choices. Implementation studies evaluating nutritional adequacy, acceptability, feasibility, and long-term sustainability are therefore warranted.

## 4. Conclusions

The six contributions to this Special Issue illustrate different dimensions of the relationship between plant-based nutrition and metabolic health. Most investigate cardiometabolic risk factors, cardiovascular outcomes, MASLD, or food environments rather than directly testing prevention of MetS. Their findings suggest that dietary quality, food preparation, and implementation context warrant attention, but they do not establish that adopting any broadly defined plant-based diet prevents MetS. This distinction is also important when interpreting broader meta-analyses on MetS, whose results differ across definitions and designs [1,2].

The findings also emphasize the importance of combining epidemiological, mechanistic, and intervention research with practical implementation strategies. Future studies should prioritize robust study designs, clearly defined dietary exposures, and clinically relevant outcomes to strengthen the evidence base and support effective dietary recommendations.

Ultimately, promoting plant-rich eating should not focus exclusively on reducing animal-derived foods, but on improving overall dietary quality and access to nutritionally adequate choices. Clearly distinguishing vegetarian, vegan, and broader plant-based exposures will be essential to future research and practice.

## Data Availability

No new data were created or analyzed in this study. Data sharing is not applicable to this article.
